# Detection of risk for future depression among adolescents: Stakeholder views of acceptability and feasibility in the United Kingdom

**DOI:** 10.1111/eip.13278

**Published:** 2022-02-01

**Authors:** Abigail Burgess, Syed Shabab Wahid, Katherine Ottman, Christian Kieling, Valeria Mondelli, Brandon A. Kohrt, Helen L. Fisher

**Affiliations:** ^1^ King's College London, Social, Genetic & Developmental Psychiatry Centre Institute of Psychiatry, Psychology & Neuroscience London UK; ^2^ Department of Psychology and Human Development, UCL Institute of Education University College London London UK; ^3^ Division of Global Mental Health George Washington University Washington District of Columbia USA; ^4^ Department of Psychiatry Universidade Federal do Rio Grande do Sul Porto Alegre Brazil; ^5^ Child & Adolescent Psychiatry Division Hospital de Clínicas de Porto Alegre Porto Alegre Brazil; ^6^ King's College London, Department of Psychological Medicine Institute of Psychiatry, Psychology & Neuroscience London UK; ^7^ National Institute for Health Research Mental Health Biomedical Research Centre South London and Maudsley NHS Foundation Trust and King's College London London UK; ^8^ ESRC Centre for Society and Mental Health King's College London London UK

**Keywords:** adolescent, depression, qualitative research, mass screening, risk

## Abstract

Aim: Depression is one of the most common mental illnesses globally and a leading cause of disability. It is often established by late adolescence and thus identifying which adolescents are most at risk is crucial to enable early intervention to prevent depression onset. We have previously developed a risk calculator to stratify which adolescents are at high risk of developing depression and in this study explore the views of stakeholders to ascertain the acceptability and feasibility of implementing such a tool in the UK.

Methods: Semi‐structured interviews were conducted with 60 UK‐based stakeholders (12 healthcare workers, 12 social workers, 12 school workers, 12 policymakers and 12 parents). Interviews were audio‐recorded and transcribed verbatim. Transcripts were analysed drawing on framework analysis techniques in NVivo 12.

Results: Six overarching themes were identified: facilitators of acceptability; barriers to acceptability; role of stakeholders in implementing risk screening; feasibility of delivering the risk calculator in practice; barriers to implementation; and policy and system implications of using it in the current UK health and social care climate. The implementation of a depression risk calculator in the UK was seen as largely acceptable and feasible by most respondents. There was a strong emphasis on the utility of schools to implement this risk calculator, although it was recognized that training and support would be essential.

Conclusions: Stakeholders were generally positive about utilizing a tool to screen for risk of future depression among adolescents in the UK but raised important concerns which should be taken into account before implementation.

## 1 INTRODUCTION

According to the World Health Organization (WHO), mental health conditions account for 16% of the global disease burden in adolescents aged 10–19 (WHO, 2019). In the United Kingdom (UK), one in 10 young people have some form of diagnosable mental health condition (Mental Health Foundation, 2015), with recent data indicating that levels of depression have increased from 9% in those born in the 1990s to 15% in those born in the early 2000s (Patalay & Gage, 2019). This is particularly concerning as depression has been shown to greatly increase the risk for life‐threatening physical disorders (Prince et al., 2007) and suicide (WHO, 2014). It has also been demonstrated that up to half of all mental disorders, including depression, begin by the age of 15, with three‐quarters being diagnosed by 18 (Kim‐Cohen et al., 2003). As such, the UK Government's latest green paper on young people's mental health made the early intervention and prevention of mental disorders a public health priority to reduce morbidity and premature mortality (DoH, 2017). However, the evidence is mixed regarding whether universal programmes to prevent mental illness in young people are effective or cost‐saving (McDaid et al., 2017; Stallard et al., 2012). Therefore, it has been suggested that future research might be better directed at targeted prevention efforts for high‐risk children and adolescents (McDaid et al., 2017). Initial trials of targeted prevention programmes among adolescents at high‐risk for depression show promising results (Brent et al., 2012; Calear & Christensen, 2020) and a recent meta‐analysis found that these were more effective than universal programmes (Werner‐Seidler et al., 2021).

Screening for future risk of developing a disorder is commonly utilized in physical health care, for example screening for obesity in children (US Preventive Services Task Force, 2017). Whilst historically neglected, it is increasingly being implemented with young children to aid prevention of mental illness (Feeney‐Kettler et al., 2010), for example, screening for internalizing and externalizing problems in pre‐school children using the Behavioural and Emotional Screener (BASC‐2 BESS) (Kamphaus & Reynolds, 2007). However, there is some scepticism concerning the utility of screening for risk of mental illness as diagnoses are often not as clear cut (Banner, 2013), which could result in large numbers of false positives (Mitchell & Coyne, 2007). Incorrectly labelling a young person as being at risk for a mental illness could be particularly problematic as stigmatizing attitudes about psychiatric disorders are still common in the UK (Curtice & Ormston, 2015) and thus those deemed at high‐risk might experience shame, distress, discrimination, and social exclusion. Furthermore, previous research has suggested that existing tools are ineffective at determining future risk of mental illness (Najman et al., 2008), which may compound service providers' reluctance to implement risk screening (McDaid et al., 2017).

Recently, as part of the Identification of Depression in Early Adolescence (IDEA) international consortium (Kieling et al., 2019), we have developed a new model for predicting the future onset of depression among adolescents (Brathwaite et al., 2020, 2021; Rocha et al., 2021). This prediction model, or ‘risk calculator,’ combines easy‐to‐obtain social and demographic information about young adolescents to predict their individual risk of developing depression at age 18. The model was originally developed and tested in a Brazilian sample but has been shown to have satisfactory performance in a UK sample (Rocha et al., 2021). However, given the above‐mentioned concerns regarding risk screening for mental illnesses, it is crucial to carefully consider the issues that might arise in using such a tool to screen adolescents for depression risk before attempting to implement it within the UK context.

Therefore, this study aimed to investigate the acceptability and feasibility of implementing screening for future risk of depression among adolescents in the UK. Qualitative interviews were conducted with a wide range of key stakeholders likely to be involved in its implementation, including health, social care and education service providers, policymakers, and parents of young people.

## 2 METHODS

This research formed part of a multi‐site qualitative study conducted in Nigeria, Nepal, Brazil, and the UK, with the aim of informing global and local policy to improve the early identification and prevention of depression in early adolescence (Wahid et al., 2020). The overall purpose of the multi‐country qualitative study was to elicit stakeholders' opinions on the feasibility and acceptability of integrating (1) screening for risk of developing depression in adolescence and (2) preventative interventions into health, social and educational settings in each country. Topic guides, types of respondents, codebook development, coding, and analysis were harmonized across sites. The current analyses focus on the UK qualitative component.

### 2.1 Participants

A purposeful, convenience sampling method was used to recruit stakeholders from London, UK. We aimed to recruit from the following groups: (i) healthcare workers (including psychologists, psychiatrists, Child and Adolescent Mental Health Services [CAMHS] workers, General Practitioners); (ii) social workers; (iii) school workers (including teachers, school counsellors, school nurses); (iv) policymakers from across the health, social care and education sectors; and (v) parents of young people. Participants were recruited using various strategies depending on their background. Policymakers and professionals were identified via social media, professional networks, and links with National Health Service (NHS) institutions. Parents were recruited via local networks, social media, schools, and advertisements in prominent areas of the local community (e.g., libraries, workplaces). All participants had to be aged between 18 and 65 years, resident in the UK and fluent in English, with current or prior experience of working with or caring for children under the age of 18.

Based on recommendations for identification of meta‐themes in multi‐sited cross‐cultural research, 20–40 respondents per country may be needed for thematic saturation across sites (Hagaman & Wutich, 2017). Therefore, our preliminary sampling goal was 45 participants per country. For each UK‐based stakeholder group, we *a priori* targeted 12 respondents as previous research has suggested that this sample size is sufficient for the identification of themes within homogenous groups (Guest et al., 2006). Because five stakeholder groups were targeted for the UK, our sample target was 60 participants. We adopted this approach as it provided a practical solution for planning the number of participants to be recruited in order to maximize the chances of obtaining sufficient data to achieve thematic saturation. However, we acknowledge that this is a complex and contentious concept (see Sebele‐Mpofu, 2020) and by no means guaranteed that we would achieve thematic saturation.

### 2.2 Procedure

All participants who met the inclusion criteria were provided with an information sheet in advance of their interview and asked to provide written informed consent. Ethical approval was granted by the Psychiatry, Nursing and Midwifery Research Ethics Subcommittee at King's College London. In‐depth semi‐structured interviews were conducted by AB (research assistant) in person or over the telephone with each participant and lasted for between 45 min and 2 h (averaging 1 h). The audio recordings of the interviews were transcribed verbatim and anonymised.

### 2.3 Topic guides

The topic guide covered three main areas: the identification of risk for depression in adolescence, facilitators and barriers of different platforms to identify risk and intervene, and system improvements. It was developed in collaboration with qualitative research teams in each of the participating countries, with country‐specific adaptations when needed. For topic guide adaptation, we took an iterative approach, with six preliminary interviews conducted with an *a priori* deductive interview guide. The resulting data were then analysed with an *a priori* deductive codebook developed based on existing literature and theory (see Wahid et al., 2020). For example, we drew upon the social ecological model of health (McLeroy et al., 1988) and the world system theory on the social origins of disease (Baer et al., 2013) to guide the development of codes to understand the role of individual, interpersonal, institutional, community and policy factors, and their interrelations, in depression risk and identification in adolescence. During this analysis, the interview guide and codebook were modified to include context‐specific questions and inductively created codes for subsequent interviews (see Supplementary Materials and Table S1).

Based on preliminary interviews in these countries, a prototype risk calculator was created for participants, modelled after similar risk calculators for diabetes and using some of the domains in the IDEA risk calculator (Rocha et al., 2021), During the interview, the mock calculator could be used to demonstrate what was meant by a risk calculator and thus gain more in‐depth answers in relation to the implementation of risk screening in practice. Participants were instructed that this was just a mock‐up and no data were collected when viewing the form.

### 2.4 Data analysis

Data analysis was conducted in coordination with parallel studies conducted in Nigeria, Nepal, and Brazil (Wahid et al., 2020). Here we focus on the results from the UK site. A systematic and progressive method, based on Framework Analysis (Smith & Firth, 2011), utilizing a combination of deductive and inductive approaches (employing Grounded Theory techniques; Strauss & Corbin, 1990), was used to understand the cultural and contextual considerations for each site. The protocol for the data collection and analysis was published in advance (Wahid et al., 2020).

During data collection, debriefing forms and analytic memos were completed by the interviewer to maintain a contextualized, reflective account of the interviews and to assist in development of inductive codes, whilst also noting methodological decisions in relation to the use of the topic guides in practice. Following preliminary coding, the constant comparison method was used to refine themes until thematic saturation was reached (Strauss & Corbin, 1990). The interview transcripts were analysed by the interviewer (AB) using NVivo‐12 software and consensus agreement was reached with HLF.

## 3 RESULTS

### 3.1 Sample characteristics

Of the 60 participants interviewed, there were 12 healthcare workers, 12 social care workers, 12 policymakers (eight from healthcare or social work; four education‐related), 12 school workers, and 12 parents/caregivers. Fifty‐one provided demographic information (see Table S2 in Supplementary Materials): the majority were female (*N* = 42, 82%) and identified as being White or White British (*N* = 46, 90%), which is reflective of the larger professional populations this sample was recruited from (Department for Education, 2018; NHS Digital, 2019) and the interviewer's own characteristics. There was some heterogeneity in age (range 20–65 years), although more participants were aged 30–39 (*N* = 20, 39%) than any other age, again reflective of the London‐based context from which this sample was recruited (ONS, 2011).

### 3.2 Observed themes

**FIGURE 1:**
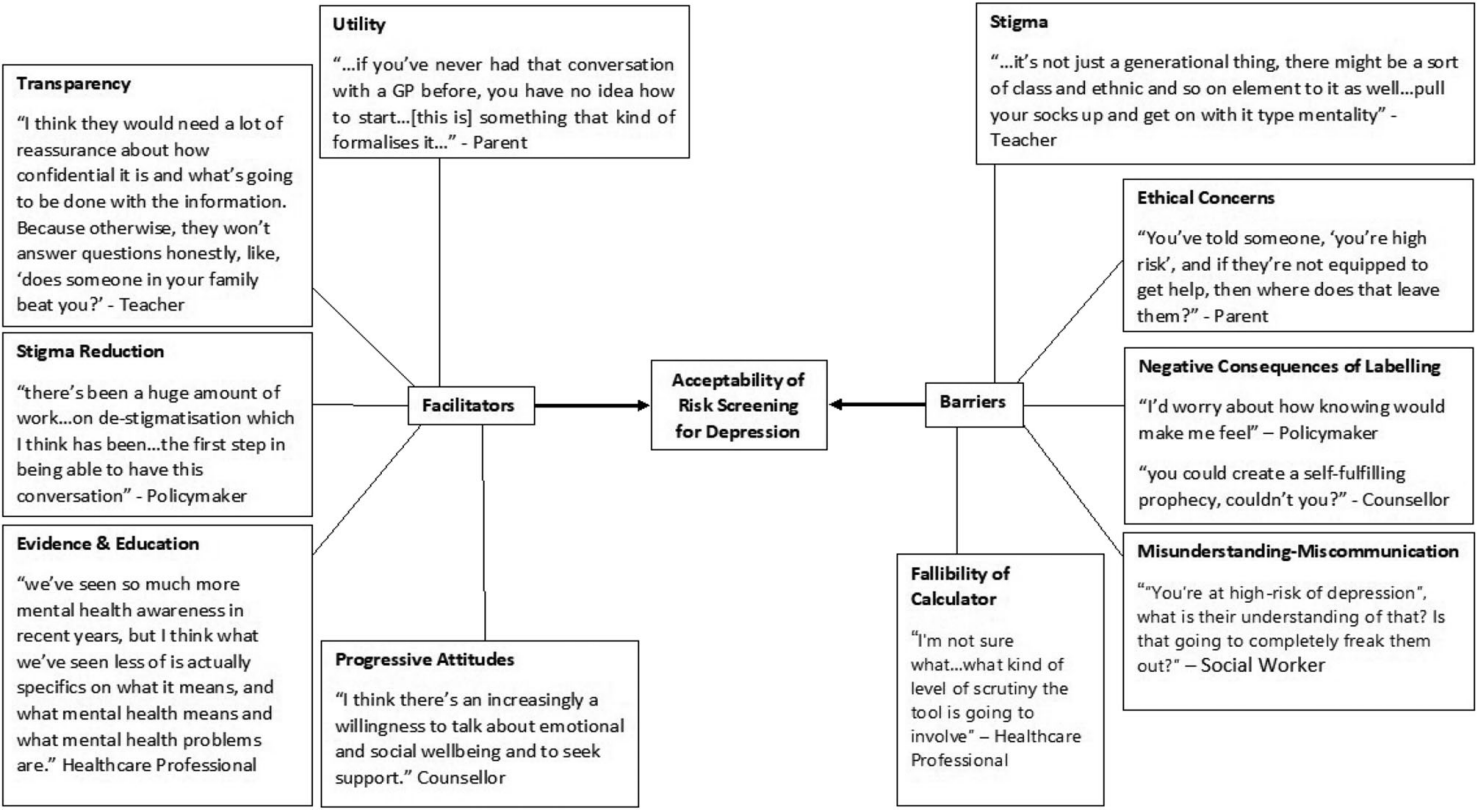
Thematic map depicting the main themes across all stakeholder groups for the acceptability of implementing risk screening for future depression among adolescents in the UK

Figure 1 displays a thematic map of the overarching themes with exemplar quotes from participants, relating to the acceptability of risk screening for depression among adolescents. Ten subthemes were identified across all stakeholder groups, within two main themes: facilitators of acceptability; and barriers to acceptability. Table 1 provides full details of these themes and subthemes (plus five additional subthemes common only in some stakeholder groups), the frequency of occurrence in each stakeholder group, and illustrative quotes.

**TABLE 1:** Themes observed in interviews with healthcare workers (H) (*n* = 12), social workers (S) (*n* = 12), related policymakers (H&S Pm) (*n* = 8), school workers (Sch) (*n* = 12), related school policymakers (Sch Pm) (*n* = 4), and parents/caregivers (P) (*n* = 12) regarding the acceptability of screening for future risk of depression in the UK

Theme	Sub‐theme	Explanation	*N* (%)	Illustrative quotes
Facilitators	Utility	Respondents described the potential usefulness of the risk calculator to provide: uniformity among professionals in terms of how to identify those at risk for depression; a means of reliably gathering information on adolescents' home lives to inform pastoral care in schools; a formal way of broaching a difficult topic; as well as highlighting how prevention can have a knock‐on effect for other societal issues.	8 (67) H 6 (50) S 4 (50) H&S Pm 9 (75) Sch 3 (75) Sch Pm 7 (58) P	“*I always appreciated when I did get information about students' home lives, and I knew what was going on. That made my life a lot easier…So, in that sense, it would be really useful to have something that, you know, was evidenced based and you did not have to do your own kind of qualitative interpretation of the data*” UK‐039 [Sch] *“…if you are already suspecting on your own that you are not doing too well mentally and you could probably use some support, if you have never had that conversation with a GP before, you have no idea how to start it without just necessarily feeling coming in like you are just going to be rejected out of hand? And something that kind of formalizes it as like no, this is just part of being human, this is one thing we check in*” UK‐082 [P]
	Mindful delivery	Respondents described that risk screening could be seen as a way to apportion blame, and therefore a sensitive approach would have to be taken for it to be acceptable.	6 (50) H 7 (58) S 0 (0) H&S Pm 7 (58) Sch 3 (75) Sch Pm 7 (58) P	“*I think if this is a screening tool, I do think parents, a lot of parents would feel like this is kind of a blame tool. If it comes out as high risk, are you saying it's my fault, kind of thing. I do think a lot of parents would not want to go there*.” UK‐028 [S] “*…making sure parents are on board. I think that's the crucial thing because if you do not communicate with parents constantly, they are going to get, they are going to be far less happy and actually, probably, because I think a lot of them would be really pleased that this measure is being put in place*.” UK‐085 [Sch] “*More supportive and less of a, less feeling of guilt if that comes up?”* UK‐017 [P]
	Transparency	Respondents (especially school workers) explained that being transparent about the outcome of the risk screen will improve its acceptability. Although, parents were, for the most part, not of the opinion that parental consent would be necessary, they did explain the importance of being transparent in terms of what the tool was going to offer their child if it is to be acceptable to them.	3 (25) H 5 (42) S 2 (25) H&S Pm 6 (50) Sch 2 (50) Sch Pm 2 (17) P	“*…there would have to be a very clear purpose. And very clear rationale for why we are investing in this and what we are going to do with that information. And who uses the calculator*” UK‐020 [Sch] “*…you would need to be more honest and upfront about the purpose of it, in the beginning. If it's a personal tool, then I would think it should be that. If it's a screening tool, then it should be clearly labelled, obviously, as a screening tool*.” UK‐066 [P]
	Option to opt out	Some respondents especially school workers) felt there would always be some who will not approve of this risk calculator and that this may be best addressed by ensuring the right to withdraw consent in a similar manner to sex education.	1 (8) H 4 (33) S 2 (25) H&S Pm 9 (75) Sch 1 (25) Sch Pm 4 (33) P	*“…you know like sex education, young people, parents can withdraw consent for that. Could they withdraw consent for filling in a form, or whatever it is, and giving them that autonomy then, whereas the majority of parents would accept for their child to go through that, some would not. You would be covering more children than we are now*.” UK‐027 [S]
	Progressive attitudes	Respondents felt attitudes towards mental health are much less stigmatizing than they used to be, particularly in the younger generation, which would improve the risk calculator's acceptability.	3 (25) H 5 (42) S 3 (38) H&S Pm 6 (50) Sch 3 (75) Sch Pm 6 (50) P	“*And one of the things I find so refreshing about my kids is how openly they talk about it. I do, you know, compared to when I was that age, you know, you would not speak, it was just a taboo topic, mental health. So, I do think we are in so much of a better place today than we were then.”* UK‐077 [H & S Pm] “*…that kind of next generation are so much more, it's so much more feasible to talk about mental health now*.” UK‐019 [P]
	Stigma reduction	Respondents (especially healthcare workers) discussed the positive impact that awareness campaigns have had on reducing mental health stigma, explaining that more awareness will help to facilitate the acceptability of screening, although school workers talked more of the need to reduce stigma among parents in order to facilitate acceptability.	8 (67) H 4 (33) S 3 (38) H&S Pm 4 (33) Sch 0 (0) Sch Pm 2 (17) P	“*I think there could be some resistance for some parents, who again, coming back to that stigma I suppose. Why are you assessing my child for risk of depression? But I think that's about wider education and people around mental health, and I think there's a lot of work being done around that, is not there? About raising the profile and you know, taking away that stigma …I think it would just be about educating parents, really*.” UK‐012 [Sch] “*I think it's just about being more aware of what depression is and just continuing that message about depression, not stigmatize it*.” UK‐017 [P]
	Evidence and education	Respondents also explained that if a sufficient evidence‐base and education were provided in support of the tool, then it would largely be acceptable in UK society.	9 (75) H 8 (67) S 5 (63) H&S Pm 9 (75) Sch 0 (0) Sch Pm 6 (50) P	“*I think if the research is there to back it up, to say that this is an accurate tool, then yeah, I think it's helpful*.” UK‐028 [S] “*I think some accurate literature about what that means that's simple, to the point, you know, no jargon and all that, but just in layman's terms would be helpful to accompany something like that to give to parents and alleviate some of their fears around that. So that if they were to come out high risk, that there is support that is available, and it's not just for assessing as high risk and then off you go, but that actually, it's about intervening early and getting that support in place to avoid them getting to a crisis point.”* UK‐012 [Sch]
Barriers	Negative consequences of labelling	Respondents were concerned that screening for risk of developing depression before clinical thresholds for diagnosis are met might risk pathologize normal life experiences, especially during the difficult teenage years when young people are often searching for an identity to latch onto, perhaps creating a self‐fulfilling prophecy.	10 (83) H 10 (83) S 1 (13) H&S Pm 7 (58) Sch 3 (75) Sch Pm 7 (58) P	“*Well, same as any screening tool. You know, you get false positives, … You have the pathologisation of somebody who does not need to be pathologized, particularly at the age and state that they are at—that it's better to have a narrative which is just about life circumstance than there's something wrong with you.”* UK‐021 [H] “*I do not know whether it might push you down that, I do not know if it's self‐fulfilling prophecy, well, that's what I've been told, it's going to happen, so that's kind of the path that you end up going down*.” UK‐012 [Sch]
	Ethical concerns	Some respondents were also concerned that there is not enough service provision to deal with those who already have depression and thus unlikely to be any support available for those at risk once they had been identified which would be unethical.	6 (50) H 7 (58) S 5 (63) H&S Pm 7 (58) Sch 2 (50) Sch Pm 6 (50) P	*“…why on earth, if I was GP, would I want to screen for stuff because I cannot even get the people with severe depression and significant self‐harming a CAMHS appointment?*” UK‐021 [H] “*I would be really concerned that simply highlighting risk of depression without providing students with a means to mitigate that risk, … it raises probably more problems than it solves*” UK‐044 [Sch]
	Exacerbation of inequalities	A small number of respondents expressed concerns that the use of a risk calculator would serve to exacerbate existing inequalities as a result of bias, and therefore would not be acceptable to use in practice with real world implications.	2 (17) H 1 (8) S 2 (25) H&S Pm 1 (8) Sch 3 (75) Sch Pm 1 (8) P	*“…on one hand, yes, you want to kind of help those that are marginalized and make sure that they have got access to all of the resources that everyone else has, and the support that they need, but on the other hand, you are doing them a disservice by singling them out and saying well you are this, so you need that, you know*.” UK‐079 [S] “*I understand there might well be a statistical correlation but there is a slight squeamishness that it might end up labelling people from certain socioeconomic classes*” UK‐039 [Sch]
	Adult anxieties	Respondents discussed that professionals are quite wary of asking such direct questions of children due to their own anxieties of finding things out that they would rather not know which might be a barrier to the acceptability of risk screening. And further to this, some parents discussed concerns about the way in which such stark questions may reflect on their parenting.	8 (67) H 1 (8) S 3 (38) H&S Pm 8 (67) Sch 1 (25) Sch Pm 5 (42) P	*“… the issue [with childhood trauma] actually tends to be more the concern of practitioners in asking these questions, or in giving the kind of inventories for them to complete. Because they have been sort of worried about the children getting upset about it. But to be honest about it, I think that more of this is in the minds of the practitioner than in the young person themselves*.” UK‐064 [H&S Pm] *“…if they would disclose anything about maltreatment…I would worry a bit myself in terms of, well, how that reflects on me as a parent*” UK‐011 [P]
	Fallibility of risk screening	Some respondents were concerned about the accuracy of the risk calculator, which may pose a barrier to its acceptability as this undermines its utility. Parents in particular were concerned that it was very brief and was particularly focused on environmental risk factors, as opposed to family history or personality traits.	5 (42) H 8 (67) S 4 (50) H&S Pm 4 (33) Sch 3 (75) Sch Pm 7 (58) P	“*Only you are reliant on the information that you are given, aren't you? So, some people who are, you know, anxious, are going to sort of put in what they need to get that high‐risk thing to try and get the service…And then you are obviously going to get others where you are not going to get the right data because they do not want you to know there's something going on, so you are always going to get the anomalies aren't you*.” UK‐023 [S]
	Miscommunication‐misunderstanding	Some respondents discussed the difficulties that can occur when parents do not understand what an assessment means, and thus they may misinterpret a high risk as being blamed for their child's problems due to the content of the questions. Parents, however, were concerned that their children may misunderstand the questions, thus throwing their parenting into doubt unnecessarily.	4 (33) H 3 (25) S 2 (25) H&S Pm 5 (42) Sch 0 (0) Sch Pm 4 (33) P	*“…often it's a misunderstanding or a misbelief, or a feeling of blame, or that we are blaming them for what happened and why their child is like that*” UK‐027 [S] *“…if they would disclose anything about maltreatment, or so, there would not be anyone to kind of help them or explain the question or what does this mean. I think I would worry a bit myself in terms of how that reflects on me as a parent*.” UK‐011 [P]
	Prior experience	Respondents discussed the idea that parents who have had poor experiences in the past with professionals may not be willing to accept any intervention, and therefore risk screening will not be acceptable to them.	5 (42) H 2 (17) S 1 (13) H&S Pm 4 (33) Sch 1 (25) Sch Pm 2 (17) P	*“…if parents have got their own mental health issues and they have not had the treatment, or say they have gone under the radar, or if they have had a bad experience of it, then they'd be thinking ‘oh, well I went there, my kid's not going there’*.” UK‐005 [H]
	Stigma	Respondents explained that there is still a lot of stigma related to mental illness which would make risk screening unacceptable due to the label it might create. School workers and parents, in particular, discussed the cross‐cultural differences in stigma among parents and families.	10 (83) H 7 (58) S 6 (75) H&S Pm 6 (50) Sch 2 (50) Sch Pm 7 (58) P	“*…there's a cultural edge to some of it for some parents, of it, you know, actually, academic results are important, mental health is not. For some of them I think there probably is a bit of a stigma attached to mental health issues. I think for some it's a kind of a denial. I do not want to be responsible for this issue, so, it's nothing to do with us as parents, it must be something to do with the school.”* UK‐045 [Sch]

All respondents, regardless of group, discussed the utility of the risk calculator at a similar frequency but were also concerned about the potential negative consequences of labelling, how accurate the tool was likely to be, and ethical concerns about data usage and lack of available services for those identified as at‐risk. These included, for example, the potential for human error in the administration of the risk tool, as well as the application of population‐level adverse childhood experience items at an individual level (Kelly‐Irving & Delpierre, 2019), and latterly, the potential for identifying adolescents at risk without having the resources to then implement effective prevention work. However, despite the reservations of some service providers about the accuracy of the risk calculator, most respondents were of the opinion that something is required to identify those at risk, and that this tool is a step in the right direction.

At least a quarter of each group felt more progressive attitudes among young people would increase acceptability of the tool, though some flagged the need for more stigma reduction among parents and greater education generally about what being ‘at‐risk’ means to facilitate acceptability. For instance, concerns were voiced about the potential for being given an ‘at‐risk’ label resulting in a fatalistic attitude to the development of problems in the future and thus that being identified as being ‘at‐risk’ would bring similar levels of stigma as a psychiatric diagnosis would. As such, some stakeholders drew similarities between the risk calculator and genetic testing, such as negative consequences on the psychological well‐being of individuals identified as at‐risk of a future disorder (Botkin et al., 2015). Respondents in our study indicated the risk calculator may serve as a defeatist self‐fulfilling prophecy for some young people and were distrustful of making definitive predictions in mental health contexts.

All groups, except healthcare and social work policymakers, felt that sensitive delivery of the tool would be important for its acceptability, meaning that it was presented in such a way as to avoid blaming the young person or their parents for the results, while parents were less concerned about transparency of the tool than the other groups. Transparency was discussed by professionals as requiring the tool to be delivered with an explicit understanding of what might or might not happen next, and the extent of confidentiality regarding any disclosures resulting from the tool. Several healthcare and school workers expressed reluctance about asking some of abuse‐orientated questions in the risk calculator to children, due to the potential safeguarding repercussions they would be responsible for initiating, whereas this was less of a concern for social workers.

**FIGURE 2:**
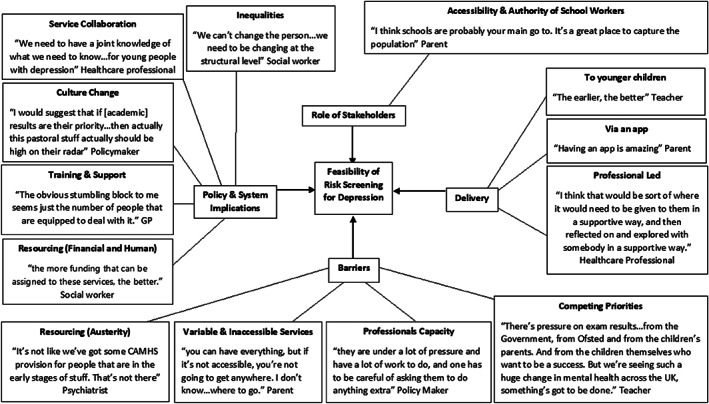
Thematic map depicting the main themes across all stakeholder groups for the feasibility of implementing risk screening for future depression among adolescents in the UK

Figure 2 displays a thematic map of the overarching themes common to all the stakeholder groups and exemplar quotes from participants, relating to the feasibility of implementing risk screening for developing depression with adolescents in the UK. Thirteen subthemes were identified across all stakeholder groups, within four overarching themes: role of stakeholders in implementing risk screening; delivery of the risk calculator; barriers to its implementation; and policy and system implications resulting from its implementation. Full details of these themes and subthemes (plus 12 additional subthemes common only in some stakeholder groups), the frequency of occurrence in each stakeholder group, and illustrative quotes are provided in Table 2.

**TABLE 2:** Themes observed in interviews with healthcare workers (H) (*n* = 12), social workers (S) (*n* = 12), related policymakers (H&S Pm) (*n* = 8), school workers (Sch) (*n* = 12), related school policymakers (Sch Pm) (*n* = 4), and parents/caregivers (P) (*n* = 12) regarding the feasibility of implementing screening for future risk of depression in the UK

Theme	Sub‐theme	Explanation	*N* (%)	Illustrative quotes
Role of stakeholders	Accessibility and authority of school workers	Most respondents felt that school workers are best placed to screen for risk of depression as they are most frequently in contact with the child, and are able to identify a range of risk factors that may not be apparent to other stakeholders until clinical threshold for symptoms has been reached.	10 (83) H 8 (67) S 7 (88) H&S Pm 10 (83) Sch 3 (75) Sch Pm 8 (67) P	“*I mean certainly form tutors are certainly the most likely people to spot things, because you see someone day in day out…Often you might have been with someone for years, so you can see someone develop and you can spot things. So, I think form tutors are probably the front line, and then, I mean, I guess heads of year are often the gatekeepers to other services.”* UK‐039 [Sch]
	Insufficient contact with healthcare workers	Respondents explained that, in the adolescent age group, GPs and other health professionals are not well placed to deliver risk screening due to the nature of their interactions with young people, coming into contact with health services largely only when there is a problem, and for a limited amount of time meaning that they do not know the young person very well.	11 (92) H 6 (50) S 0 (0) H&S Pm 2 (17) Sch 1 (25) Sch Pm 6 (50) P	“*I'm thinking more maybe parents or teachers…I'm not sure about healthcare professionals. Because I'm not sure that they will have come to health professionals before*” UK‐097 [P] “*…it's so patchy…I was really lucky I had an amazing GP…But…I know other people that have got GPs that just come out with ludicrous things…GPs that just have not got a clue about anything outside of their remit*” UK‐019 [Sch]
	Poor perceptions of social care workers	Respondents discussed the idea that although social workers are very well placed in terms of accessing the child in their family home, they may not be the best stakeholder group to deliver screening due to poor perceptions of their role.	5 (42) H 7 (58) S 1 (13) H&S Pm 4 (33) Sch 0 (0) Sch Pm 0 (0) P	“*To be honest, I think it's quite fundamental in the sense that the relationship between social services and communities is quite poor, in relation to children. I think in terms of families, a lot of the clients of social workers, you know, are actively frightened by social services. And I think that's a really fundamental problem, really*.” UK‐047 [H&S Pm]
	Parents: position, knowledge, conflicted	Respondents talked about parents being well placed to identify risk of developing depression, providing collateral information to professionals, but equally that they may be part of the problem and so are not as well placed as professionals to identify risk. Parents also acknowledged that those without personal experience of depression might be less able to identify their child was at risk.	8 (67) H 5 (42) S 2 (25) H&S Pm 8 (67) Sch 1 (25) Sch Pm 12 (100) P	*“…you might automatically, you might say, well obviously the parents, the people that know the children or the individuals very well, but then often they can be not the best people because they see them every day in different contexts and you know, you have kind of the complexity of the relationship and maybe certain emotional issues come into play, so it can be quite difficult*.” UK‐079 [Sch] *“But most parents probably would not have that knowledge, or you know, even know what to do with it if they did recognize it.”* UK‐019 [P]
	Peers: supportive and supported	Respondents discussed the important role that supportive peers play in adolescents' lives, but that in order to be able to support risk screening, they would have to be supported themselves.	7 (58) H 6 (50) S 2 (25) H&S Pm 9 (75) Sch 2 (50) Sch Pm 9 (75) P	“*I think peers are absolutely key, because they are the ones, they know each other better than we as professionals will ever probably know that young person. You know, they know, they'll notice a difference in their presentation that's quite subtly, that perhaps a class teacher might not with 30 other children in the room.”* UK‐012 [Sch] *“…they kind of have to be kind of facilitated in that because, kind of, saying that you are worried about a friend is quite a difficult thing to do, I think, sometimes, as well because you do not want to be disloyal or anything.”* UK‐008 [H]
	Diffused responsibility	Respondents explained that whilst some stakeholders are better placed than others, it should be everyone's responsibility to identify risk of developing depression in a similar way to safeguarding.	8 (67) H 8 (67) S 1 (13) H&S Pm 6 (50) Sch 1 (25) Sch Pm 5 (42) P	“*Yeah, so obviously lots of people could contribute to this, but…I do not think it's necessary to identify a particular person or a particular role*” UK‐044 [Sch] “*I think any…any place that a child has contact, should have that in their mind set*.” UK‐005 [H]
Delivery	Direct approach	Health and social care professionals described the simple and direct approach as being more feasible because it is not a time‐consuming or knowledge intensive exercise. Other respondents explained that whilst uncomfortable, it is important that the risk calculator is direct and simple so that children can answer important questions to get them the help they need.	5 (42) H 5 (42) S 4 (50) H&S Pm 4 (33) Sch 0 (0) Sch Pm 8 (67) P	“*I think the questions were very direct. And I think they need to be. I think there's a lot of, I do not know how to word it, but it's, I think people are scared of talking about mental health sometimes aren't they? And I think that direct questioning approach was really good…they are quite closed questions were not they, yes or no. So, yeah, which I thought was good.”* UK‐012 [Sch]
	To younger children	Some respondents felt it may be more beneficial to do the screening in healthcare settings with younger children, as a universal screen, because this is where it can be most feasibly integrated along with other universal interventions before it is too late to intervene successfully. Similarly, school workers and parents suggested screening in schools might be more feasible for younger children as there are better relationships between teachers and children in smaller classes and they can be supported more effectively.	7 (58) H 4 (33) S 3 (38) H&S Pm 3 (25) Sch 2 (50) Sch Pm 7 (58) P	“*GPs. Health visitors. I think health visitors are key because they are doing the outreach for the under 5 s, so they are seeing risk factors start early on, so that maybe through this work could be troubleshooted before they get to school. That's where the change needs to happen, in the first five years.”* UK‐001 [S] “*…it might even be easier as a primary school teacher because you are with the same 30 children every day, for 5 days a week, whereas I can imagine it's a bit harder for secondary school teachers when they are not seeing them so frequently*” UK‐079 [Sch]
	Via an app	Respondents were of the opinion that the most feasible method of delivery for a risk calculator was using apps as these are both accessible and sustainable.	8 (67) H 8 (67) S 2 (25) H&S Pm 9 (75) Sch 1 (25) Sch Pm 4 (33) P	“*I think something like that in an app would be brilliant. And as I was going through it, I was thinking that, that that would lend itself to being an app, that, you know, young people are all about apps, aren't they? Everything's about smart phones and all that. And I think it's just honing into what they are interests are. Like, if that was on a piece of paper, it feels very different than something that's quite interactive.”* UK‐012 [Sch]
	Professional‐led	Respondents were largely of the opinion that risk screening would be best delivered by a professional to provide support to the more vulnerable children answering potentially difficult questions.	10 (83) H 10 (83) S 3 (38) H&S Pm 10 (83) Sch 4 (100) Sch Pm 10 (83) P	“*But I'm wondering if it's something that you stumbled across and then sort of came out as being high risk of depression, and you do not quite have the confidence, or the skill set to follow up on any of these. I'm being overcautious, but…I suppose some of the questions, yes, I think for teens, I think…it ought to be sort of done in a kind of safe space with someone who they can then kind of go to rather than you doing it in your bedroom and coming out with this risk score and then not feeling quite sure what to do with it.”* UK‐055 [P]
	Self‐directed	Respondents felt the risk screening could be feasibly completed by young people themselves, and the anonymity via use of technology could encourage honest answers from young people. However, they also raised concerns regarding confidentiality and how, when and where the information divulged would be shared.	10 (83) H 9 (75) S 5 (63) H&S Pm 10 (83) Sch 1 (25) Sch Pm 10 (83) P	“*I think I'd kind of lean towards them filling it in themselves directly, just because there is some sensitive information for when you are a teen…Where it's important to get an honest answer and it's really, really unlikely you'd get an honest answer if someone else was looking over their shoulder or filling in answers*.” UK‐082 [P] “*…it [social media] might create…the sense of a safe space for people to disclose things they might otherwise not say face to face. It might even facilitate greater disclosure of risk.”* UK‐007 [H]
	Support infrastructure	Respondents discussed the need for a support infrastructure to facilitate the delivery of an online risk calculator—without which, its safe application in practice was considered less feasible.	9 (75) H 9 (75) S 5 (63) H&S Pm 10 (83) Sch 1 (25) Sch Pm 10 (83) P	“…*let us say, you get a young person, they fill it in, and they come up to mum or dad and say ok I've filled this in and it thinks I'm depressed. Well, if parents are not ok with that, …it's about kind of making sure that they get access to somebody to talk to that about, so kind of taking that information into somebody in school, or talk to their friend, or you know, giving them links of where else they can help and support from…”* UK‐006 [S]
	Universal versus targeted	Respondents explained that those who are most likely to need risk screening are likely to be the ones who are not accessing it. They thought that universal screening may be the best way to overcome this issue, with parents likening it to a vaccination programme.	8 (67) H 6 (50) S 6 (75) H&S Pm 1 (8) Sch 1 (25) Sch Pm 5 (42) P	“*Something that kind of gets parents on board, erm, in a way that does not get a lot of knee jerk resistance to it? Kind of like vaccinations have developed…And in a way that just makes it a part of looking after your health, just a sort of part of your preventative care, you know? Get your five a day, get your mental health screening, you know?”* UK‐082 [P]
Barriers	Resources (austerity)	Many respondents were concerned about the lack of resources to feasibly implement risk screening. Respondents described the deficit in current mental health service provision, which was unfunded and unable to meet current demand, meaning that there is no capacity for support in the event of early identification of risk.	9 (75) H 8 (67) S 4 (50) H&S Pm 9 (75) Sch 1 (25) Sch Pm 6 (50) P	“*It's not like we have got some CAMHS provision for people that are in the early stages of stuff. That's not there. So why on earth…would I want to screen for stuff because I cannot even get the people with severe depression and significant self‐harming a CAMHS appointment?”* UK‐021 [H]
	Variable and inaccessible services	Respondents discussed concerns that services vary depending on locality and budget, and were inaccessible to young people who may not know where to go and the system is not able to go to them, which may be barriers to implementing risk screening more broadly.	8 (67) H 6 (50) S 6 (75) H&S Pm 9 (75) Sch 2 (50) Sch Pm 6 (50) P	“*I think that the sort of support that people access can be very variable, depending on what's available in the area. So, I know some GPs have direct links with, sort of, mental health, psychology, psychiatry services, but some GPs do not have that access at all. So, I think the actual provision can be very, very poor*.” UK‐009 [H] “*Because the trouble is, as a young person, I would not have known where to go. … I think unless you are told really explicitly what is available, and people are willing to listen to you, you are probably not likely to reach out*.” UK‐023 [S]
	Competing priorities	Respondents discussed concerns that there are other priorities above preventing mental illness in the UK more broadly, but also within the education system which might make it less feasible to implement risk screening in practice.	8 (67) H 7 (58) S 2 (25) H&S Pm 9 (75) Sch 3 (75) Sch Pm 6 (50) P	“*It's a difficult one, because you can imagine, you have got so much pressure to meet the Ofsted targets and meet the exam results, where's the motivation to put it [risk screening] in?*” UK‐074 [H]
	Professionals' lack of capacity	Respondents also described schools as being over‐burdened by a growing amount of responsibility for child welfare, which is not currently adequately resourced in their budgets which focuses on reaching education targets.	12 (100) H 10 (83) S 2 (25) H&S Pm 10 (83) Sch 3 (75) Sch Pm 8 (67) P	“*But it's not their fault. They just have not got the resources and the time to do anything about it, you know. They have to concentrate so much on these results that there's no time to look at individuals*.” UK‐019 [P] “*I think schools are completely over‐worked and overwhelmed and are taking on more and more of a therapeutic role, and resources need to go into that*.” UK‐001 [S]
Policy & Systems Implications	Culture change	Respondents discussed the need for a change in the culture of health and social care delivery, as well as in schools, which is currently focused on reactive work as opposed to proactive prevention to embrace wellbeing and strengthen trusting relationships between staff and young people.	9 (75) H 9 (75) S 4 (50) H&S Pm 8 (67) Sch 4 (100) Sch Pm 8 (67) P	“*…the NHS is very good at keeping people alive, it's not very good at keeping them well, or preventing them from getting unwell (laughs), you know, it's always a firefight response*.” UK‐024 [H] “*It's really about trust. It's really about trust. And that's so difficult to create that environment, I think, for them*.” UK‐018 [P]
	Top‐down change	Respondents explained that in order to enact change and implement risk screening for depression, it would be important to get policy makers on board to filter changes down to front line workers, by making the economic case for it.	3 (25) H 8 (67) S 7 (88) H&S Pm 4 (33) Sch 2 (50) Sch Pm 2 (17) P	“*I think that if you are really serious about this and you are serious about the mental health of our young people and about our society, this has to be a top down approach. This has to come from a policy perspective, first of all.”* UK‐079 [Sch Pm] “*Policy makers, obviously, really important, because they are the ones that can ultimately drive the change*.” UK‐049 [H&S Pm]
	Service collaboration	Respondents discussed the need for services to join up to provide both consistency of care, and to share learning to try and target the inequalities seen in the currently disjointed system.	7 (58) H 4 (33) S 4 (50) H&S Pm 5 (42) Sch 1 (25) Sch Pm 2 (17) P	“*For me, it is something about linking people up because, I do not know, sometimes it feels like someone's referred to here and there and places, so just having…everyone using similar tools or similar interventions or similar strategies and ideas about it would be helpful*.” UK‐003 [H] “*I would like to see more joining up of services because I think that's something that has really let my son down*.” UK‐018 [P]
	Service user involvement	Respondents explained that in order to improve the system in the UK, it is integral to listen to and act on the perspectives of young people in order to ensure that policies actually serve the needs of the people they are designed for.	4 (33) H 3 (25) S 3 (38) H&S Pm 3 (25) Sch 2 (50) Sch Pm 3 (25) P	“*I think they—I think someone from health probably needs to sit down with these people and sort of say this is what people were exposed to, young people were exposed to, and get young people working on that. You know, I think there is something about getting them involved*.” UK‐005 [H]
	Inequalities	Respondents discussed disparity in terms of societal inequalities, explaining that in delivering risk screening, it is necessary to be mindful of systemic issues so that blame is not located in the individual but sustainable change is made by changing the system.	6 (50) H 4 (33) S 1 (13) H&S Pm 2 (17) Sch 2 (50) Sch Pm 3 (25) P	“…*we cannot change the person, you know—if you are changing the personal level, then you are never going to make sustained changes. So…we go in and we sort of change the individual family, well we do some work with the individual family, and then we go away and then it comes back. That's not what needs to change. We need to be changing at the structural level*” UK‐054 [S]
	Resourcing	All respondents discussed the importance of funding in improving the system in the UK. Respondents voiced concerns about the impact of austerity on current service provision and the need for not only money, but also adequately trained staff to be recruited to frontline services to meet demand.	8 (67) H 2 (17) S 2 (25) H&S Pm 6 (50) Sch 1 (25) Sch Pm 2 (17) P	“*Well, certainly, I think there's lots of steps that can be taken. Not least, sort of funding into mental health services, and…also other services that provide sort of care, or mental health work—so be that social services or primary care services, or secondary care services as well. I think funding has a lot to do with it, and the more funding that can be assigned to these services, the better resourced, the better placed they will be to sort of access and support young people.”* UK‐009 [H]
	Training and support	Respondents emphasized the need for training and support in order for professionals (especially teachers) to feel confident to deliver the risk calculator to young people, with health and social care workers suggesting support be delivered like clinical supervision.	8 (67) H 8 (67) S 3 (38) H&S Pm 12 (100) Sch 3 (75) Sch Pm 2 (17) P	“*I guess you would need… training in terms of like how to phrase things… some kind of instructions, or not a manual but information about how best to deliver it. And almost how to phrase things or introduce it kind of thing. If that makes sense?*” UK‐065 [P] *“In terms of finding support for them going through the process, we have supervision where we can discuss with our managers all the work that we are doing.”* UK‐028 [S]
	Research	Respondents, largely those working in health and social care, explained that the best way to drive policy change is to fund and engage in research to begin to understand the scale of the problem, and the utility of screening and prevention for risk of developing depression.	6 (50) H 3 (25) S 5 (63) H&S Pm 1 (8) Sch 0 (0) Sch Pm 2 (17) P	*“…this could be one off research definitely, but if we incorporate it into the system, you know, we need to show kind of figures possibly. I think that would help, that if you nip it in the bud, this is how much you are saving the NHS or recouping resources later on…I think that's how they count is not it?”* UK‐029 [P]

Stakeholders from all groups consistently identified schools as being the most feasible and accessible platform from which to deliver screening to the largest number of young people. Notably, there were broad similarities between all respondents in terms of the barriers to feasibility, with most discussing a lack of resources (i.e., money for training, and staff time to devote to its implementation) given chronic understaffing in existing services. Moreover, respondents were concerned that schools are increasingly being used as a place to take on more pastoral care than the system is currently competent to manage and that teachers are ill‐equipped to deal with mental health concerns.

One way in which stakeholders discussed overcoming these barriers was to deliver risk screening via an app, which was deemed to be the most accessible and cost‐effective way of identifying children at risk. Frontline service providers were keen that the use of apps was professionally led, whilst policymakers were keen that there was sufficient infrastructure regulating the backroom functioning of such an app, ensuring that adequate referral policies are in place before widespread implementation. Additionally, whilst most respondents highlighted that screening should be overseen by professionals, it was acknowledged that there is insufficient capacity at present to actually do this. Many therefore suggested the most feasible solution is for adolescents to complete the tool themselves. However, across all groups there was a recognition that culture change is needed across the relevant institutions to facilitate the implementation of risk screening, for example moving from a reactive to a proactive stance in health and social care contexts.

## 4 DISCUSSION

To our knowledge, this is the first study to investigate, with a range of key stakeholders, the acceptability and feasibility of implementing screening for future risk of depression among adolescents within the UK context. We found that the majority of stakeholders interviewed in this study suggested that risk screening for developing depression is both acceptable and feasible in the UK, with many citing its utility for filling a gap in service provision that currently fails to prevent young people's mental health from deteriorating to a crisis‐point. However, a number of caveats and important issues for consideration were also raised regarding the implementation of such screening.

Stigma was a concern impacting acceptability of risk screening for developing depression. Being labelled as being ‘at‐risk’ of a mental illness in adolescence can be both beneficial in order to facilitate access to support, as well as harmful as a result of the negative reactions of their peers (Welsh & Brown, 2013). This age group may be particularly at risk of stigmatization from peers (Crosnoe et al., 2008). These views are consistent with recent criticisms that diagnosing individual‐level vulnerabilities from probabilistic, population‐based tools is inherently problematic and may serve to exacerbate inequalities rather than serve those who are most vulnerable to future problems (Kelly‐Irving & Delpierre, 2019).

Schools were highlighted as being the most feasible setting to implement risk screening. However, despite schools being widely thought of as the most accessible place to support the majority of children (Hanley et al., 2017), only 2% of secondary schools currently have a published policy relating to mental health (DfE, 2018). Indeed, a report by the Mental Health Foundation (2018) found that 73% of teachers felt mental health was not given sufficient priority during their training. This echoes the concern raised by many respondents that more investment would be needed to skill‐up staff to implement depression risk screening and ensure clear pathways were in place to access preventive interventions.

The idea that apps should be delivered under the supervision of a professional until efficacy and safety is better established is consistent with previous research which suggests that professionals are cautious when referring individuals to online resources because of concerns regarding their effectiveness (Sinclair et al., 2013). At least in the pre‐pandemic era, there was limited evidence concerning the efficacy of smartphone apps in children and young people's mental health care (Hollis et al., 2017) and this potential approach for risk screening thus requires careful investigation.

### 4.1 Limitations and future research

It is important to note that within the qualitative interviews, some respondents mistook the nature of the risk calculator to be something that was being used to identify current symptoms of depression rather than those at high risk of developing depression in the future, which may have impacted upon the findings obtained. However, the iterative nature of the interview topic guide, and the use of a prototype risk calculator to demonstrate its potential use in practice to respondents may have improved the depth and specificity of our findings. The participants in this study were, however, a relatively homogenous group (predominantly white and female) who are not representative of the wider population in the UK, thus limiting the generalizability of the findings. Unfortunately, we were unable to recruit adolescents into our study due to ethical restrictions within the UK. Future research would benefit from investigating the attitudes of adolescents themselves towards risk screening as these may be affected by generational shifts in attitudes towards mental health as discussed by the respondents in this study. It may also be useful to investigate the effectiveness of stigma reduction campaigns and the next steps required to improve de‐stigmatization of mental health issues to increase the wider acceptability of such a risk screening tool.

## 5 CONCLUSION

Risk screening for future development of depression in adolescence was seen by most respondents as largely acceptable and feasible in the UK. This may be because of widespread anti‐stigma campaigns, such as the Time to Change campaign (a social movement led by the UK‐based charities ‘Mind’ and ‘Rethink Mental Illness’), and a growing recognition of the issues facing young people, placing prevention nearer the top of the public, political and clinical agendas. Although the opinions of stakeholders were largely positive regarding the risk calculator, a lack of resources was identified as a major barrier to its feasibility in practice. This is reflective of wider concerns within mental health care in the UK, where CAMHS are recognized as reaching only 35% of those in need (CQC, 2018), and even fewer of those who may require preventative support. Clear referral pathways and accessible services would need to be in place before implementation of risk screening for future depression among adolescents could be considered. Subsequently, research will be required to develop a physical or online screening tool based on our predictive model and test its implementation potential in school settings in the UK.

## CONFLICT OF INTEREST

Dr. Mondelli has received research funding from Johnson & Johnson, a pharmaceutical company interested in the development of anti‐inflammatory strategies for depression, but the research described in this paper is unrelated to this funding. All other authors declare they have no conflicts of interest to report.

## Supporting information


**Appendix** **S1**: Supporting Information

## Data Availability

The data that support the findings of this study are available on request from the corresponding author. The data are not publicly available due to privacy or ethical restrictions.
