# Supplementary material for: Detection of risk for future depression among adolescents: Stakeholder views of acceptability and feasibility in the United Kingdom
Source: Early Interv Psychiatry. 2022 Feb 1;16(12):1319–34. doi: 10.1111/eip.13278 (PMC10078769; doi:10.1111/eip.13278)
Supplement: Supplementary file 1 — Appendix S1: Supporting Information [file EIP-16-1319-s001.docx]

**Supplementary Material**

*Burgess et al. “Detection of risk for future depression among adolescents: Stakeholder views of acceptability and feasibility in the United Kingdom”*

**Semi-Structured Interview Topic Guide**

**Identification of Risk of Depression in Adolescence**

- In your institution, have you heard of a tool being used to screen for risk of developing depression?
- In your institution, who could be assigned to identify the risk of developing depression in adolescents?
- Which groups of people do you think are the best positioned to recognise the risk of developing depression in adolescents?
  - E.g. families/peers/community health care workers/specialists/social workers/teachers/religious leaders/police

**Introduce mock risk calculator (to be referenced throughout)**

- What is your initial response to the risk calculator?
  - Length of questionnaire, acceptability/appropriateness of questions, usability
- Who do you see using this calculator, and where or when would they use it?
  - Adolescents? Parents? Teachers? Healthcare worker?
  - Feasibility
- Do you foresee any challenges for using this questionnaire? If so, how might we address them?
  - Are there any potential negative consequences of using something like this?
  - What solutions or changes would your recommend for these challenges?
  - Do you see any benefits to using this calculator?
- What additional resources or instructions would need to go along with such a calculator?
  - What additional information would you like to know for someone who was high risk?
  - Low risk?
- Are there any risks in stigmatising adolescents who are identified as being at risk of developing depression?
  - How can these risks be mitigated?
  - Who can be involved to help in this process?
- How can we best leverage technology (mobile phones; internet; social media; etc.) to help identify adolescents at risk of developing depression?
  - What are the major facilitators of using these technologies?
  - What are the major barriers of using these technologies?
  - Do you have a solution to overcome these barriers?

**Facilitators and Barriers of Different Places/People to Identify Risk and Facilitate Interventions**

**Home and Family Environment:**

- How can the home and family environment be best used as a place to identify risk of developing depression early among adolescents?
  - What are the major facilitators of using the home environment?
  - What are the major barriers of using the home environment?
  - Do you have a solution to overcome these barriers?
- What role can the family play in facilitating early interventions to prevent adolescents developing depression?
  - What might be some of the challenges for families?
  - How might we overcome these challenges?

**School:**

- How can schools be best used as a place to identify risk of developing depression early?
  - What are the major facilitators of using the school environment?
  - What are the major barriers of using the school environment?
  - Do you have a solution to overcome these barriers?
- What role can teachers play in facilitating early interventions to prevent adolescents developing depression?
  - How feasible would it be to incorporate that into their current role?
  - What might be some of the challenges?
  - How might we overcome these challenges?

**Peers:**

- How are peers placed to identify risk of developing depression early?
  - What might be some of the challenges for peers?
  - How might we overcome these challenges?
- What role can peers play in facilitating early interventions to prevent adolescents developing depression?

**The Community:**

- How can community groups and community centres be best used as a place to identify risk of developing depression early?
  - If struggling – why probe
  - What are the major facilitators of using community groups?
  - What are the major barriers of using community groups?
  - Do you have a solution to overcome these barriers?
- What role can community leaders (e.g. religious) play in facilitating early interventions to prevent adolescents developing depression?
  - How feasible would it be to incorporate that into their current role?
  - What might be some of the challenges?
  - How might we overcome these challenges?

**Healthcare:**

- How can primary care facilities, outpatient clinics, specialty care facilities be used as places to identify risk of developing depression early?
  - What are the major facilitators of using such facilities?
  - What are the major barriers of using such facilities?
  - Do you have a solution to overcome these barriers?
- What role can [community] health workers play in facilitating early interventions to prevent adolescents developing depression?
  - How feasible would it be to incorporate that into their current role?
  - What might be some of the challenges?
  - How might we overcome these challenges?

**Other Public Sectors:**

- How are social workers placed to identify risk of developing depression early?
  - How feasible would it be to incorporate that into their current role?
  - What are the barriers?
  - Do you have a solution to overcome these barriers?
- What role can social workers play in facilitating early interventions to prevent adolescents developing depression?
- How are the police placed to identify risk of developing depression early?
  - How feasible would it be to incorporate that into their current role?
  - What are the barriers?
  - Do you have a solution to overcome these barriers?
- What role can the police play in facilitating early interventions to prevent adolescents developing depression?

**NGOs:**

- What role can NGOs play in identifying risk of developing depression in adolescents?
- What role can NGOs play in facilitating early interventions to prevent adolescents developing depression?

**System Improvements to Identify Risk and Prevent Depression in Adolescents**

- In the UK what steps need to be taken to create a better system that can identify risk of developing depression and prevent it?
  - How feasible would it be to create this system?
  - Which groups of people should be involved in this system? What would their roles be?
  - What are some of the existing facilitators for such a system?
  - What types of resources would be needed?
  - What types of trainings need to be provided? Who needs to be trained?
  - What would be some of the biggest challenges?
  - What would your advice be towards overcoming these challenges?
- If a system like this were to be developed, would be it be acceptable in UK society?
  - In the family, community, school environment, including teachers, policy-makers etc.?
  - Would it be acceptable if teachers and school workers were trained to identify adolescents at risk for depression?
  - Would it really be something that would provide help where it is needed?
- Are there any risks in increasing stigma for adolescents during and/or after receiving early interventions?
  - How can these risks be mitigated?
  - Who can be involved to help in this process?

**Table S1**

UK specific inductive code book

| Umbrella Topic | Parent Code | Parent Code Description | Child Code | Child Code Description |
| --- | --- | --- | --- | --- |
| Feasibility of Risk Screening for Depression | Role of Stakeholders | Apply this code to extracts where the roles of various stakeholders are discussed in terms of the feasibility of implementing risk screening. These can include: parents, peers, GPs, school workers, social workers, health care professionals | Accessibility & Authority of School Workers | This code has been applied where extracts discuss school workers as in a good position to implement screening because they have a lot of easy access to children and are professionals which means they are accountable and able to be skilled up to deliver it in a safe way |
|  |  |  | Accessibility & Authority of Healthcare Professionals | This code has been applied where extracts discuss healthcare professionals’ role, generally that healthcare workers are inaccessible but do have authority in healthcare matters (Including knowledge & skills which are useful) |
|  |  |  | Accessibility & Authority of Social Care Professionals | This code has been applied where extracts discuss social care workers role, generally that social care professionals often have unprecedented access to home life, but that the authority of social workers regarding safeguarding and care proceedings impedes the relationship with families who are often scared of them |
|  |  |  | Position, Knowledge & Detachment of Parents | This code has been applied where extracts discuss the role of parents who have been described as very well placed in terms of access, but not so well placed because they may lack knowledge and are not able to detach their own emotions regarding the potential for it being their fault/them being problematic |
|  |  |  | Supported & Supportive Peers | This code has been applied where extracts discuss the role that peers play, including that when supportive they are a great resource because adolescents are closer to their peers than anyone else during their search for identity & belonging, but that they need supporting to do a good job |
|  |  |  | Diffused Responsibility | This code has been applied where extracts discuss the idea that identifying risk of developing depression is everybody's job - a bit like safeguarding. |
|  | Delivery | Apply this code to extracts where the delivery modes of risk screening for depression is discussed. These can include; online or in-person formats, administration by a professional or adolescent alone, targeted versus blanket screening. | Via an App | This code has been applied where extracts discuss the delivery of risk screening via an app - generally lauded for inclusivity and accessibility |
|  |  |  | To Younger Children | This code has been applied where extracts discuss the need to deliver risk screening to much younger children, partly because primary schools are more supportive and therefore may be more feasible to deliver risk screening in and partly because the teenage years are difficult years when children are hard to access anyway, also includes the concept that the younger the better in terms of prevention being effective |
|  |  |  | Professionally Led | This code has been applied where extracts discuss the need for professionals to deliver risk screening because it has to be supported (not safe to leave children hanging by themselves) |
|  |  |  | Self-Directed | This code has been applied where extracts discuss the idea that completing it alone will lead to more honest answers because young people will be less concerned about divulging private information to strangers and the implications of that - distinction in cases of lived experience parents. |
|  |  |  | Direct Approach & Simple | This code has been applied where extracts discuss the concept that the calculator is direct and simple, does not ask too many/too complicated questions which is good and necessary because you only get a straight answer by asking a straight question and also that it has to be simple to be engaging and accessible for young people |
|  |  |  | Universal vs Targeted | This code has been applied where extracts discuss the merits of delivering risk screening to a universal population (e.g. at school/GP) as opposed to a targeted population (e.g. only once concerns were raised or in special educational needs schools) |
|  | Barriers | Apply this code to extracts where barriers to the feasibility of implementing risk screening are discussed. These can include; lack of adequate service provision, lack of human resources, issues with accessibility, issues with the target population, lack of education and training, barriers to delivery methods (e.g. social media being part of cause as well as the solution) | Resourcing (Austerity) | This code has been applied where extracts discuss the idea that implementing screening is not so feasible currently because there are no resources due to austerity to deliver it and also as a consequence, that there are no resources to support child afterwards which means that even if it were delivered in a resource-lite fashion that it wouldn't be feasible to support people after the fact which makes it unethical - linked below |
|  |  |  | Variable & Inaccessible Services | This code has been applied where extracts discuss the idea that services are currently very variable in quality (geographic dependent - better public services in poorer areas because more funding but equally, higher SES groups able to move to these areas which then makes them inaccessible) and also inaccessible (no one really knows what's out there and isn't able to access the stuff that is there because waiting lists are so incredibly long and also services are inflexible - do not come to the SU and the SU is not able to go to them always) which means that implementing screening is not so feasible everywhere/won't reach everyone |
|  |  |  | Professionals Capacity | This code has been applied where extracts discuss the idea that professionals are incredibly overworked, overwhelmed, and undereducated about mental health whilst simultaneously having more responsibility for children's welfare than ever before - also to some extent includes comments more broadly on professionals roles in the context of barriers to implementation (e.g. being inflexible, 'not my job' kind of attitude) |
|  |  |  | Competing Priorities | This code has been applied where extracts discuss the idea that there are other greater priorities than preventing mental health for the people in charge including Brexit but more so about schools prioritising grade above wellbeing due to Ofsted and national frameworks |
|  | Policy & System Implications | Apply this code to extracts where the ways in which the system may have to change in order to make risk screening feasible in practise are discussed, or ways it will change if risk screening is successfully implemented are discussed. These can include; funding and investment to resource it, linking up of services, training/education deficits, and the use of screening | Service Collaboration | This code has been applied where extracts discuss the need for services to join up to share knowledge about children as well as about mental health and to improve the accessibility of services for young people so that, for example, the GP will know as much about their situation as school will as the hospital will etc. - singing from the same hymn sheet in effect |
|  |  |  | Culture Change (Proactive not Reactive, Relationship building, Inequalities (MH parity with PH) | This code has been applied where extracts discuss the need for a culture change to take place, in reference to schools this often refers to building relationships to improve the relationships between teachers and pupils (trust, pastoral care etc), otherwise includes the need for a culture change from reactive to proactive approach, for mental health (MH) to have parity with physical health (PH) especially in relation to policy to acknowledge/reduce inequalities - mostly concerning objection to conservative government ideologies/approaches/policies |
|  |  |  | Service User Involvement | This code has been applied where extracts discuss the need for service users, especially young people, to be involved in the formulation and delivery of the risk calculator in order for them to actually do it |
|  |  |  | Top-down Change | This code has been applied where extracts discuss the need for policy makers and funders to be brought on board because fundamental change effectively occurs from the top down, leadership is key in changing cultures as opposed to the bottom up changes that are discussed like people changing their attitudes and approach personally |
|  |  |  | Resourcing (Financial & Human) | This code has been applied where extracts discuss the need for money and more staff to be able to deliver anything effectively, starting with the services they are currently supposed to be delivering, let alone anything new like risk screening |
|  |  |  | Training & Supervision | This code has been applied where extracts discuss the need for better training and more supervision if risk screening is going to work, especially in relation to teachers - some talk of introducing mental health training to teacher training programmes |
|  |  |  | Research | This code has been applied where extracts discuss the need for more research to determine what interventions work because we don't know enough about it and whilst risk screening is a handy first step to identify the problem at hand, largely thought that we don't know enough about the what next step is/should be |
| Acceptability of Risk Screening for Depression | Facilitators | Apply this code to extracts where ways in which the acceptability of risk screening can be facilitated are discussed. These can include: taking a sensitive approach to avoid stigma, ensuring there is a sufficient evidence base prior to implementation, ensuring an equitable method of consent | Mindful Delivery | This code has been applied where extracts discuss the need to deliver risk screening sensitively, avoiding blaming anyone, parents or the young people themselves, for the result in order for it to be acceptable |
|  |  |  | Consent to Withdraw | This code has been applied where extracts discuss the idea that giving parents and children completing it the option to withdraw from it would make it more acceptable (like sex education) - interestingly, teachers more concerned with this than parents |
|  |  |  | Transparency (Implications) | This code has been applied where extracts discuss the idea that in order to be acceptable, you have to be extremely transparent in delivering it especially in terms of what happens next, things around confidentiality and safeguarding responsibility for example |
|  |  |  | Evidence & Education | This code has been applied where extracts discuss the idea that a lot of evidence supporting the efficacy of the tool and education around it and the why people should do it, as well as the fundamentals of what depression actually is would make it more acceptable both to the people who will be delivering it as well as the people being asked to complete it - also education is described as the next step now that we've started raising awareness because awareness alone isn't enough |
|  |  |  | Stigma Reduction | This code has been applied where extracts discuss the positive effects of stigma reduction campaigns and also the need for more/to keep going with the high-profile campaigns, to make this acceptable |
|  |  |  | Utility | This code has been applied where extracts have discussed the idea that implementing risk screening is acceptable because it will be useful, both as a formal avenue to talk about difficult topics and also as a way to get information from children that would otherwise not be known because no one asks these kinds of questions so directly, and also that something is needed because we have a big problem - gap in the market kind of thing. Also discussed as a way to standardise service provision - get everyone on the same page across sectors and also to provide data about the prevalence of the problem to help research efforts |
|  |  |  | Progressive Attitudes | This code has been applied where extracts have discussed the idea that attitudes towards mental health have moved on significantly in this country in the last 20 years in this country and so it will be acceptable to do it, especially with young people who are very willing to talk about things like this |
|  | Barriers | Apply this code to extracts where issues that might prevent the risk calculator being acceptable are discussed. These can include: the futility of screening, ethical concerns relating to use of data or the questions themselves | Exacerbating Inequalities | This code has been applied where extracts have discussed the idea that risk screening is not acceptable because ACEs are problematic and that labelling people as at risk of depression is just exacerbating existing inequalities |
|  |  |  | Stigma (Cross-cultural, Intergenerational & Gendered) | This code has been applied where extracts have discussed the idea that due to cultural and gender differences and older people being more stigmatising towards mental health, it might not be acceptable to certain groups of people/society - e.g. boys more stigmatised, still don't talk as freely |
|  |  |  | Ethical Concerns (Service Provision & Data Processing) | This code has been applied where extracts discussed the idea that there is just no service provision for CAMHS in this country and that identifying young people at risk of something we can't help them with is unethical. This code has also been applied where extracts have discussed the idea that there are ethical concerns in relation to data processing - where will it be stored, by who, who will use it - however this is much less well filled out than the service provision aspect |
|  |  | Negative Consequences of Labelling | Self-fulfilling Prophecy | This code has been applied where extracts have discussed the danger that labelling someone as at high risk would create a self-fulfilling prophecy whereby they think they will develop it so they go ahead and act like it, thereby reducing the acceptability of screening |
|  |  |  | Pathologising Normality | This code has been applied where extracts have discussed the idea that everyone calls themselves depressed these days, and that the experiences covered in the calculator would lead to difficulties but that at is a normal reaction, there's no need to pathologise - sometimes objection to the medical model, sometimes objection to “snowflakes” |
|  |  |  | Miscommunication-Misunderstanding | This code has been applied where extracts discuss concerns that children will go home and tell their parents the wrong thing because they haven't understood the purpose or results of the calculator and then they will not be happy (e.g. my teacher told me I'm depressed now, because they don't understand the concept of risk) and also incorporates elements that parents may feel they are being blamed for it therefore get angry |
|  |  |  | Adult Anxieties | This code has been applied where extracts discuss concerns that adults don't want to be asking these kinds of questions because it opens up a whole can of worms which nobody wants to deal with so they just avoid it (from the professionals perspective) and from the parents perspective that professionals won't do it well which will just be upsetting |
|  |  |  | Fallibility of Calculator | This code has been applied where extracts discuss concerns that the calculator may not be accurate/is not to be trusted, partly because people are human and will make mistakes with it, partly because children will lie (either to get more attention or to cover up abuse they don't want to share - goes both ways), and partly because ACEs are not the only aspect to developing mental health problems also (rarely) the idea that you can't really predict or prevent it in the first place because people are individuals who make their own path. Parents particularly concerned about the brevity of the calculator - too short to be right because MH is more complex than that |
| Extraneous |  | Apply this code to chunks of data which are interesting discussion points, but not directly relevant to the feasibility or acceptability of implementing risk screening for depression. These can include, for example, where respondents talk of the need for parents to have training to better manage children's issues. |  |  |

**TABLE S2**

Demographic characteristics of the UK-based sample, broken down by stakeholder group. *Note. The total sample comprised 60 participants—12 in each group—however, only 51 respondents provided demographic information. This is reflected in the N reported in the table below.*

| Demographics | Total  % (n)  (N = 51) | Healthcare Workers  % (n)  (N = 12) | Social Workers  % (n)  (N = 10) | Educators  % (n)  (N = 8) | Policy Makers  % (n)  (N = 9) | Parents/Caregivers  % (n)  (N = 12) |
| --- | --- | --- | --- | --- | --- | --- |
| Gender |  |  |  |  |  |  |
| Female | 82 (42) | 83 (10) | 100 (10) | 88 (7) | 56 (5) | 83 (10) |
| Male | 18 (9) | 17 (2) | - | 13 (1) | 44 (4) | 17 (2) |
| Ethnicity |  |  |  |  |  |  |
| White/White British | 90 (46) | 92 (11) | 90 (9) | 88 (7) | 100 (9) | 83 (10) |
| Asian/Asian British | 4 (2) | - | 10 (1) | - | - | 8 (1) |
| Black/Black British | 6 (3) | 8 (1) | - | 13 (1) | - | 8 (1) |
| Mixed | - | - | - | - | - | - |
| Age |  |  |  |  |  |  |
| 20-29 | 14 (7) | 25 (3) | 20 (2) | 13 (1) | 11 (1) | - |
| 30-29 | 39 (20) | 33 (4) | 40 (4) | 63 (5) | 44 (4) | 25 (3) |
| 40-49 | 27 (14) | 25 (3) | 20 (2) | 13 (1) | 11 (1) | 58 (7) |
| 50-59 | 18 (9) | 17 (2) | 10 (1) | 13 (1) | 33 (3) | 17 (2) |
| 60-65 | 2 (1) | - | 10 (1) | - | - | - |
